# Erratum: A single theoretical framework for circular features processing in humans: orientation and direction of motion compared

**DOI:** 10.3389/fncom.2013.00028

**Published:** 2013-04-04

**Authors:** Tzvetomir Tzvetanov

**Affiliations:** Institut für Informationsverarbeitung, Leibniz Universität HannoverHannover, Germany

In the article *A single theoretical framework for circular features processing in humans: orientation and direction of motion compared* (*Tzvetanov, 2012, Front. Comput. Neurosci. 6:28. doi: 10.3389/fncom.2012.00028*) a presentation error occurred concerning the equations defining *a* and *w* within the text (subsection 4.4, page 9).

The sentence on page 9, subsection 4.4:

The priors on the three parameters were: *l* - beta probability distribution with parameters Beta (1.1, 20); μ - normal probability distribution with parameters Norm (*a*, 10*w*) with *a* and *w* being the mean and standard deviation obtained from each experimental data set {*x_i_, p_i_*} (*a* = ∑ *p_i_x_i_* and $w=\sqrt{\sum p_{i}{\left(x_{i}-a\right)}^{2}})$; σ - gamma probability distribution with parameters Gamma (*a_G_*, *b_G_*) fixed at *a_G_* = 1 + *w/b_G_* and $b_{G}=(\sqrt{5}-1)w/2$ such that the mode and variance of the Gamma distribution is *w*.

must be replaced by:

The priors on the three parameters were: *l* - beta probability distribution with parameters Beta (1.1, 20); μ - normal probability distribution with parameters Norm (*a*, 10*w*) with *a* and *w* being the mean and standard deviation obtained from each experimental data set {*x_i_, n_i_, y_i_*} as follows: *a* = ∑*q_i_ x_i_*, $w=\sqrt{\sum p_{i}{\left(x_{i}-a\right)}^{2}}$, and *q_i_* = *n_i_/∑_i_n_i_*; σ - gamma probability distribution with parameters Gamma (*a_G_*, *b_G_*) fixed at *a_G_* = 1 + *w/b_G_* and $b_{G}=(\sqrt{5}-1)w/2$ such that the mode and variance of the Gamma distribution is *w*.

